# Possible cross-sensitivity between sertraline and paroxetine in a panic disorder patient

**DOI:** 10.4103/0253-7613.64497

**Published:** 2010-04

**Authors:** Praveen H. Khairkar, Govind M. Bang, Adarshlata B. Singh, Prashant G. Tiple

**Affiliations:** Department of Psychiatry and Dermatology, Jawaharlal Nehru Medical College, Datta Meghe Institute of Medical Sciences, (Deemed University), Sawangi (m), Wardha, Maharashtra, India

**Keywords:** Cutaneous adverse effects, cross-sensitivity, paroxetine, sertralineIntroduction

## Abstract

Cross-sensitivity due to paroxetine and sertraline, the SSRIs, is rarely reported in the literature. We report an adverse drug reaction to paroxetine and sertraline in a patient of panic disorder, who initially developed a maculopapular, erythematous, pruritic rash in the third week with sertraline 50 mg/day. The rash resolved within 2 days of its discontinuation and oral supplementation of diphenhydramine and betamethasone. 10 days following discontinuation of sertraline, the patient was shifted on sustain release paroxetine 12.5 mg/day when another skin reaction with the same appearance and distribution appeared on day 4 of it, suggesting a possibility of cross-sensitivity, a drug class effect. This case report intends to improve the awareness among clinicians to use caution when choosing an alternative SSRIs.

## Introduction

Selective serotonin reuptake inhibitors (SSRIs) are one of the most frequently prescribed classes of drugs for the treatment of panic disorder. Only two of the SSRIs (paroxetine and sertraline) are FDA approved for the treatment of panic disorder, whereas the other SSRIs are typically used off-label.[1] Cutaneous adverse effects of SSRIs are uncommon and include mild photosensitivity, pruritus, urticaria, toxic epidermal necrolysis, Steven-Johnson syndrome, and leukocytoclastic vasculitis.[2,3] In contrast, SSRIs have also been used to treat pruritus associated with cholestasis[4] and polycythemia vera.[5] A 2 to 3% frequency of rash was reported in placebo-controlled clinical trials of paroxetine and sertraline for obsessive-compulsive disorder, panic disorder, and other anxiety disorders.[6,11] However, relative to other drug classes of psychotropic medications, minimal information is available about the frequency of SSRI-induced rash, rash characteristics, differences among SSRIs in propensity to cause a rash, and cross-sensitivity.[3,6,9] Interestingly, cross-reactivity between psychotropic drugs with similar structures has been reported in the literature;[8,9] however, cross-reactivity among SSRI antidepressants is unexpected given their differences in chemical structure. We report a case of adverse drug reaction to paroxetine and sertraline in a patient of panic disorder without agoraphobia. A computerized MEDLINE search on the above subject yielded a report of two cases of possible cross-sensitivity between SSRIs, and in both the cases paroxetine was involved.[7,8]

## Case Report

A 39-year-old female came to our psychiatric outpatient services with the recurrent transient episodic paroxysms of panic attacks, each lasting for about 10-15 min since 6 years. She had been intermittently given 0.5 mg of alprazolam and 40 mg propranolol per day by private physician with which she had minimal improvement. Her baseline chest radiograph, electrocardiography, tread mill test, thyroid function tests, and cranial tomogram of brain were found to be within normal limits. The patient did not have any past or family history of allergy or dermatological diseases. We started her on 25 mg/day of sertraline and 1.5 mg/day of clonazepam while alprazolam and propranolol were discontinued. On day 8, sertraline was increased to 50 mg/day. She perceived about 50% improvement by week 3 with the regime. However, developed maculopapular, erythematous rash especially on sun exposed areas starting cranio-caudally. The rash was generalized all over the trunk and limbs, with some facial involvement. There was no palm or sole involvement noted. Her dermatologic consultation was taken and her rash resolved within 2 days of drug discontinuation and supplementation with 75 mg/day of diphenhydramine and 6 mg/day of betamethasone. The patient again had recurrence of panic attacks within a proceeding week because of which paroxetine 12.5 mg/day was given to her following 10 days of sertraline discontinuation. Another skin reaction with the same appearance and similar distribution appeared on day 4 of paroxetine. We stopped paroxetine and no other drug from same class was further tried as the patient has refused, while clonazepam was continued along with Jacobson's progressive muscular relaxation. The patient gradually improved almost to her premorbid state and is still in remission with a periodic follow-up. No history of concomitant or recent chocolate or coffee consumption, other drug or herbal medicine intake, fever, arthralgia, or joint swelling, was reported.

## Discussion

Both the sertraline and paroxetine are FDA approved among the SSRIs for the treatment of panic disorder with sertraline, and paroxetine had equivalent efficacy in panic disorder, but sertraline was significantly better tolerated and was associated with significantly less clinical worsening during taper than paroxetine.[1] In placebo-controlled trials, the frequency of SSRI's induced rash was 3% for sertraline and 2% for paroxetine versus 2% with placebo.[10,11] In general, acute cutaneous adverse reactions due to SSRIs do not appear to be dose related, and they recur promptly on rechallenge.[10] The cross-sensitivity among them has been uncommonly reported between paroxetine and sertraline.[7,8] Our case is of particular interest because in the previous two reports rash was initially noted first on exposure to paroxetine followed by sertraline; however, reverse scenario has been seen in our patient who may have experienced an adverse drug reaction and possible cross-reactivity, since paroxetine was started after 10 days of sertraline discontinuation while the usual washout time for sertraline is about 7 days (The half-life of sertraline is approximately 25 h; and it has a less potently active metabolite with half life of 60 to 70 h).[12] The chronology of events suggested that both reactions were caused by the SSRIs, suggesting a class effect although the confirmative validative patch test could not be done due to patient's refusal. Apparently it is pointing toward the fact that cross-sensitivity between sertraline and paroxetine is much more possible than among other SSRIs in the class. Since sertraline and paroxetine are structurally different, one interpretation could be that there may be individuals who are very sensitive to increase in serotonin concentration in blood. The other speculation could be that skin adverse reactions to SSRIs may be due to high activity in the serotonergic system at the dermal and epidermo-dermal junctional area rather than a hypersensitivity to the drug molecule itself.[13,14]

## Conclusion

Given the large number of psychiatric patients receiving SSRIs, lack of published reports and results of post-marketing safety and tolerability comparisons, SSRI-induced rash appears to be a relatively rare phenomenon. Nonetheless, these reactions can occur and, as in our patient, may lead to discontinuation of drug therapy. To conclude, some index of suspicion is warranted by physicians and psychiatrists for patients who develop a skin rash during treatment with an SSRI due to the possibility of cross-sensitivity between them.
